# Metabotropic glutamate receptors—guardians and gatekeepers in neonatal hypoxic-ischemic brain injury

**DOI:** 10.1007/s43440-024-00653-x

**Published:** 2024-09-17

**Authors:** Damian Mielecki, Ewelina Bratek-Gerej, Elżbieta Salińska

**Affiliations:** Department of Neurochemistry, Mossakowski Medical Research Institute PAS, Pawińskiego 5, 02-106, Warsaw, Poland

**Keywords:** Metabotropic glutamate receptors, Neonatal hypoxia-asphyxia, HI, Therapeutic potential of mGluR agonists and antagonists

## Abstract

Injury to the developing central nervous system resulting from perinatal hypoxia–ischemia (HI) is still a clinical challenge. The only approach currently available in clinical practice for severe cases of HI is therapeutic hypothermia, initiated shortly after birth and supported by medications to regulate blood pressure, control epileptic seizures, and dialysis to support kidney function. However, these treatments are not effective enough to significantly improve infant survival or prevent brain damage. The need to create a new effective therapy has focused attention on metabotropic glutamate receptors (mGluR), which control signaling pathways involved in HI-induced neurodegeneration. The complexity of mGluR actions, considering their localization and developmental changes, and the functions of each subtype in HI-evoked brain damage, combined with difficulties in the availability of safe and effective modulators, raises the question whether modulation of mGluRs with subtype-selective ligands can become a new treatment in neonatal HI. Addressing this question, this review presents the available information concerning the role of each of the eight receptor subtypes of the three mGluR groups (group I, II, and III). Data obtained from experiments performed on in vitro and in vivo neonatal HI models show the neuroprotective potential of group I mGluR antagonists, as well as group II and III agonists. The information collected in this work indicates that the neuroprotective effects of manipulating mGluR in experimental HI models, despite the need to create more safe and selective ligands for particular receptors, provide a chance to create new therapies for the sensitive brains of infants at risk.

## Introduction

Perinatal hypoxia–ischemia (HI) typically results in permanent damage to the central nervous system, known as hypoxic-ischemic encephalopathy (HIE). The changes observed in the newborn's brain following HI are both structural and functional, potentially disrupting the brain maturation process [1].

Studies indicate that 15–25% of infants with HIE do not survive the neonatal period, and 25% of those who do survive may develop long-term complications such as cerebral palsy, intellectual disability, epilepsy, hearing or vision impairments, or learning challenges [2, 3]. Currently, therapeutic hypothermia initiated shortly after birth is the only clinically available and accepted intervention for severe cases of HI [4]. This treatment is often complemented with medications to regulate blood pressure and manage epileptic seizures and dialysis to support kidney function. However, these interventions have shown limited effectiveness in significantly improving infant survival rates or preventing brain damage [5]. Hence, there is a pressing need for novel and effective treatment approaches.

The primary mechanism underlying cellular degeneration and death post-HI, though not fully elucidated, is generally recognized [6]. HI disrupts normal energy metabolism, leading to reduced adenozyno-5′-trifosforan (ATP) production, acidosis, and following excessive release of glutamate. The elevated extracellular glutamate levels result in overactivation of various types of glutamate receptors, most of which can potentially contribute to the initiation and progression of neurodegenerative processes. HI-induced oxidative stress plays a pivotal role in the pathogenesis of neuronal damage. Under normal circumstances, cellular defense mechanisms maintain low levels of reactive oxygen species (ROS) to prevent oxidative stress. However, during HI, the excessive production of oxygen radicals overwhelms the neutralization mechanisms, resulting in toxic concentrations that impair cellular functions and damage lipid membranes, proteins, and DNA. Furthermore, impaired mitochondrial functioning leads to the initiation of apoptotic processes and neuronal death [7].

**Fig. 1:**
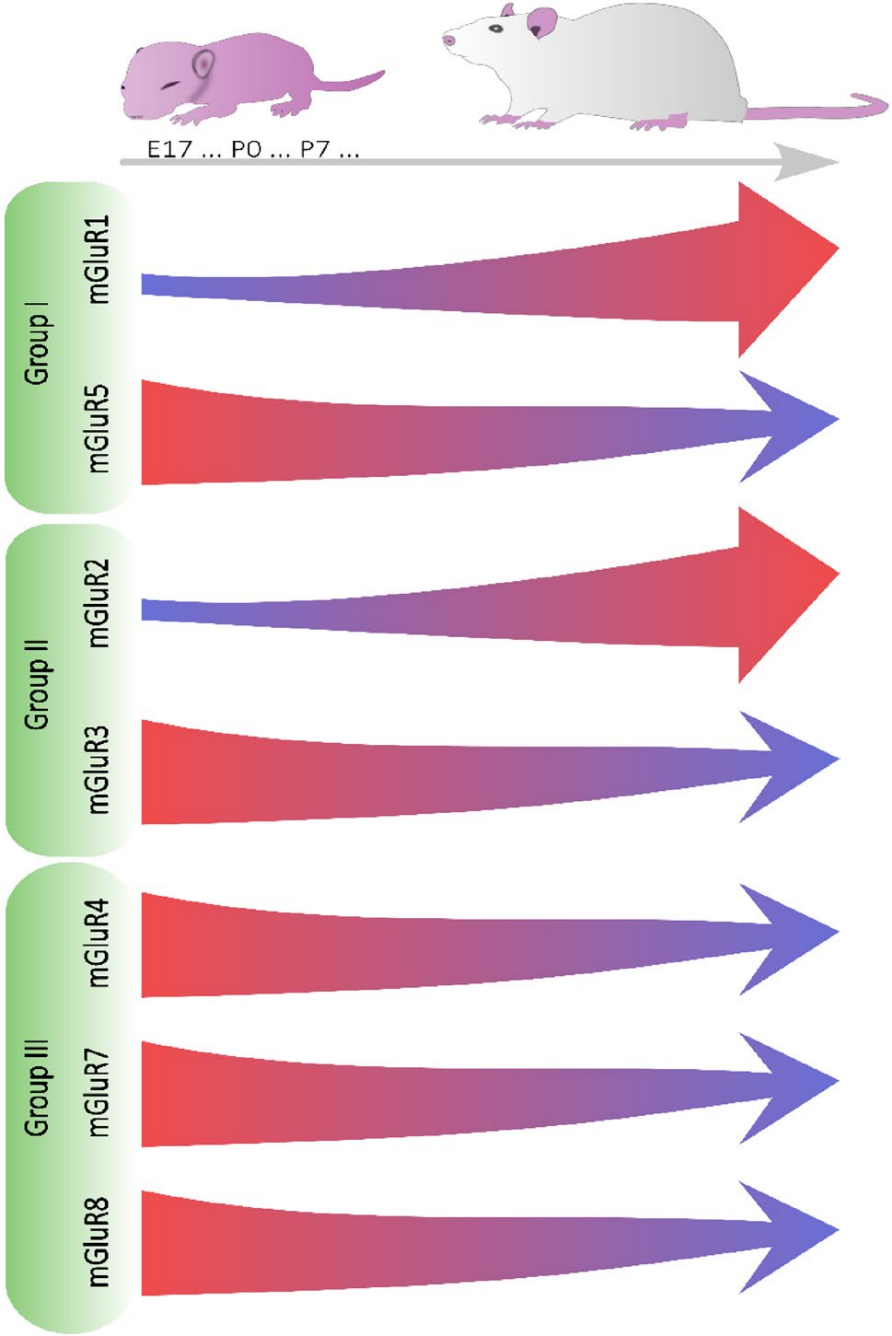
Schematic representation of the changes in metabotropic glutamate receptor (mGluR) expression in the rat brain throughout development and maturation. These changes correlate with those observed in the human brain. E17 refers to embryonic day 17, while P0 indicates postnatal day 0 (the day of birth). Arrows transitioning from blue to red indicate an increase in expression, whereas arrows shifting from red to blue represent a decrease in expression

The neurodegenerative impact of excessive glutamate release is primarily associated with ionotropic glutamate receptors, particularly α-amino-3-hydroxy-5-methyl-4-isoxazole propionic acid (AMPA) and *N*-methyl-D-aspartate (NMDA) receptors. Activation of NMDA receptors, which are coupled to a calcium ions (Ca^2+^)-permeable channels, and the subsequent massive influx of Ca^2+^ into the cell, are widely accepted as the main triggers of intracellular events leading to cellular death [8, 9]. Recent research has indicated that extrasynaptic NMDA receptors, activated by the excessive release of glutamate, play a role in promoting cell death, while synaptic receptors contribute to neuroprotective mechanisms [10]. This has focused the attention of scientists looking for new therapies on possibilities of both enhancing the effect of synaptic activity and decreasing activation of extrasynaptic NMDA receptors. Consequently, there is a growing interest in metabotropic glutamate receptors (mGluRs), which can modulate excitatory synaptic transmission [11]. Although the expression of mGluRs in the central nervous system changes during development, their widespread distribution throughout the brain underscores their significant role in its function (Fig. 1). Research on the involvement of these receptors in neurodegeneration/neuroprotection and the effects of mGluR activation have intensified over the past decade, particularly in the context of HI-induced brain damage, with increasing attention directed towards mGluRs.

mGluRs are typical G-protein coupled receptors, comprising eight subtypes categorized into three main groups (group I, II, and III) based on sequence homology, G-protein coupling specificity, and pharmacological characteristics [12].

Group I mGluRs (mGluR1 and mGluR5) are coupled with Gαq/11 proteins, which subsequently activate the β isoform of phospholipase C (PLCβ), initiating a signaling pathway that leads to intracellular Ca^2+^ mobilization and protein kinase C (PKC) activation [12]. Group II (mGluR2 and mGluR3) and group III (mGluR4, mGluR6, mGluR7, and mGluR8) mGluRs are coupled to Gαi/o proteins, whose activation inhibits adenylyl cyclase.

Moreover, mGluR activation triggers G-protein-independent signaling mechanisms that regulate ion channels and other downstream signaling partners, enhancing the potential for regulating cell metabolism under both physiological and pathological conditions [12, 13].

This review aims to present the available information on the roles of mGluR subtypes in the mechanisms of HI-induced neurodegeneration/neuroprotection. We will also explore whether modulating mGluRs with subtype-selective ligands could potentially emerge as a novel treatment strategy for birth asphyxia.

To offer a comprehensive overview, we will first delve into the specific roles and characteristics of each mGluR group in the context of perinatal hypoxia–ischemia. Subsequently, we will review preclinical studies investigating the effects of mGluR modulation in various HI models. Finally, we will discuss the potential therapeutic implications and challenges in translating these findings into clinical applications.

### Group I mGlu receptors

Activation of group I mGluRs, mGluR1 and mGluR5, stimulates PLCβ, leading to the generation of inositol-1,4,5-triphosphate (IP3) and diacylglycerol (DAG), two secondmessengers that not only participate in intracellular Ca^2+^ release and PKC activation but also activate transient receptor potential (TRP) channels permeable to Ca^2+^. Both receptors are anchored to NMDA receptors and can facilitate their activation by alleviating the Mg^2+^ blockade [11, 13]. Consequently, the excessive activation of mGluR1 and mGluR5 categorizes them as factors involved in neurodegeneration.

Studies on the expression of group I mGluRs in the developing human and rat brains have revealed that these receptors are highly expressed in neurons and glial cells during a critical period of vulnerability to HI [14, 15]. In the early postnatal phase, mGluR1 and mGluR5 can be found in proximal dendrites and the cell soma, predominantly in the extrasynaptic region of the postsynaptic membranes of dendritic spines.

Immunocytochemical studies have shown that mGluR5 predominates in the immature human and rat brains, while mGluR1 emerges in limited quantities in the rat brain towards the end of the first postnatal week [15–17]. The minimal presence of mGluR1 in early stages progressively increases during brain development, whereas mGluR5 expression diminishes [18].

Functional studies have demonstrated that mGluR1, rather than mGluR5, mediates transient calcium ions currents in rat brain neurons during early postnatal days, with mGluR5 being responsible for Ca^2+^ elevation in astrocytes [14, 19].

Most research indicates that the actions of group I mGluRs under conditions of heightened glutamate release, as seen in brain ischemia and neonatal hypoxia–ischemia, lean towards neurotoxicity [20, 21]. However, there are reports supporting the neuroprotective actions of group I mGluRs [21–23]. For instance, studies have shown that the application of (S)-3,5-dihydrocy-ohenylglycine (DHPG), an agonist of group I mGluRs, can prevent astrocytes and GABAergic neurons from ischemic damage [24]. Baskys et al. (2005) observed neuroprotective effects of a group I mGluR agonist (DHPG) on NMDA-induced cell death in organotypic hippocampal slice cultures and suggested that the observed protection was induced by activation of mGluR1, which resulted in increased neurogenesis [23].

By analyzing experiments involving a wide range of competitive and noncompetitive agonists and antagonists of group I mGluRs in various models of cerebral ischemia, Pellegrini-Giampietro concluded that mGluR1 and mGluR5 play distinct roles in ischemia-induced neuronal death, with mGluR1 primarily involved in the mechanisms leading to post-ischemic neuronal injury [25]. It was suggested that the activation of presynaptically located mGluR1 inhibits gamma-aminobutyric acid (GABA) release, and thus, the application of mGluR1 antagonists reduces ischemia-induced neurodegeneration by enhancing GABA-mediated neurotransmission.

However, later publications showed that mGluR5 also contributes to post-ischemic processes. Studies have shown that mGluR1 antagonists ( +)-2-Methyl-4-carboxyphenylglycine (LY367385)(3-ethyl-2-methyl-quinolin-6-yl)-(4-methoxy-cyclohexyl)-methanone methanesulfonate (EMQMCM), and YM-202074 reduce brain damage following focal cerebral ischemia in adult rats and forebrain ischemia in gerbils, suggesting the involvement of mGlu1 receptors in activating pathways leading to postischemic neuronal death [26–29]. Additionally, Makarewicz et al. (2006) demonstrated that an mGluR1 antagonist, EMQMCM, protected against immature brain damage in experimental birth asphyxia, contrasting with the lack of neuroprotective effects observed with a potent mGluR5 antagonist, 3-[(2-methyl-1,3-thiazol-4-yl)ethynyl]pyridine (MTEP) [29].

The potential role of mGluR5 in neuroprotection in neonatal hypoxia–ischemia remains less explored. As mentioned above, Makarewicz et al. (2006) did not observe a neuroprotective effect of the potent mGluR5 antagonist MTEP in neonatal rat HI, while a potent negative modulator, fenobam, potentiated HI-induced brain damage. However, at lower doses, fenobam significantly reduced the number of apoptotic cells [29, 30]. In the same experimental model, the authors utilized a potent positive mGluR5 allosteric modulator, (4-fluorophenyl)[(3S)-3-[3-(4-fluorophenyl)-1,2,4-oxadiazol-5-yl]-1-piperidinyl]methanone (ADX47273), but did not observe neuroprotective effects [30].

Interestingly, the application of mGluR5 antagonists or agonists has shown neuroprotective effects in focal cerebral ischemia in adult rats and global forebrain ischemia in gerbils [29, 31]. The observed neuroprotective effects of a selective mGluR5 agonist (R,S)-2-chloro-5-hydroxyphenylglycine (CHPG) application in adult rat brain ischemia was linked to its anti-apoptotic properties, contrasting with the outcomes in neonatal rats [30]. Furthermore, Riek-Burchardt and colleagues (2007) did not observe functional or histological improvements following the application of CHPG in a model of endothelin-1-induced focal cerebral ischemia in adult rats [32].

Additional differences in group I mGluRs response to brain ischemia were detected. Global ischemia in adult rats led to a significant downregulation of mGluR5 expression in the hippocampus, whereas*in utero* hypoxia–ischemia did not alter mGluR5 expression but notably reduced mGluR1 expression[33, 34].

Modulating mGluR5 in astrocytes has also been proposed as a potential method for neuroprotection after ischemia. Experiments conducted on neonatal rat hippocampal and mouse cortical slices revealed that the impairment of glutamate transporters on astrocytes and the response of GABAergic neurons to excitatory glutamate release during ischemia were prevented by the mGluR1/5 agonist DHPG [19, 24, 35]. Activation of mGluR5 in astrocytes in an optic nerve explant culture subjected to oxygen–glucose deprivation (OGD) also resulted in cytoprotection. This effect was associated with the inhibition of apoptosis mediated by PLCb and the activation of PI3K/Act, Nuclear factor erythroid 2-related factor 2** (**Nrf2), and Nuclear factor kappa B (NF-κB) pathways [36, 37]. Furthermore, activation of mGluR5 in microglia cell cultures exposed to OGD reduced cytotoxicity, ROS accumulation, and the release of inflammatory cytokines, and the effect was reversed by inhibiting the brain-derived neurotrophic factor/Tropomyosin-related kinase receptor B (BDNF/TrkB) pathway. The authors suggested that the BDNF/TrkB pathway plays a crucial role in the protective effects of mGluR5 activation [38]. Regrettably, there is a lack of data demonstrating similar effects in animal models of neonatal hypoxia–ischemia.

These findings underscore the complex role of group I mGluRs in perinatal asphyxia and emphasize the necessity for further research to unravel their potential as therapeutic targets. The divergent effects observed in neonatal versus adult models of brain ischemia, along with the varied responses in different cell types, highlight the significance of developmental stage and cellular context in determining the outcomes of mGluR modulation in hypoxic-ischemic conditions.

### Group II mGlu receptors

The group II metabotropic glutamate receptors consist of mGluR2 and mGluR3, which are expressed throughout the brain, with mGluR2 expression predominantly restricted to neurons. Both receptors are coupled to inhibitory G-protein (Gi/Go) and function to negatively regulate adenylyl cyclase, thereby inhibiting cAMP signaling [39].

The distribution of mGluR2 is more restricted compared to mGluR3 [40]. mGluR2 receptors are predominantly located in presynaptic regions of neurons, distanced from the neurotransmitter release site [41], while mGluR3 is expressed in both pre- and postsynaptic regions of neurons outside the synapse, as well as in glial cells. When situated in presynaptic membranes, mGluR2/3 inhibit neurotransmitter release from glutamatergic, GABAergic, and neuromodulatory presynaptic terminals, whereas postsynaptic mGluR3 has been reported to enhance excitability by inhibiting potassium currents and activating calcium currents [42–44]. In glial cells, activation of mGluR3 upregulates the expression of glutamate transporters, enhancing glutamate uptake from the synapse [45, 46].

During the early postnatal period (PN1-PN25), both mGluR2 and mGluR3 are expressed, albeit with distinct developmental trajectories. Initially, low expression of mGluR2 in the first postnatal days significantly increases in the first two weeks of life, while the highly expressed mGluR3 at birth decreases over the first few weeks [47, 48]. These alterations in mGluR2/3 expression suggest their pivotal role in brain development and neuronal network maturation, indicating their potential significance in various developmental brain disorders.

The unique characteristics of mGluR2/3 have garnered significant attention in neuroscience research. Specifically, their ability to inhibit glutamate release from presynaptic terminals and modulate various signaling pathways in postsynaptic terminals of neurons and glial cells has positioned them as potential drug targets for neurodegenerative and neuropsychiatric diseases. Moreover, their potential to suppress the excessive glutamate release induced by ischemia has made agonists of these receptors promising drug candidates in the treatment of brain ischemia and birth asphyxia.

However, initial research outcomes were varied. Early experiments with the use of selective group II agonists did not demonstrate neuroprotective effects in rat model of permanent ischemia or focal ischemia in gerbils [49, 50]. Intriguingly, some studies reported a highly protective effect of a selective mGluR2 negative allosteric modulator (ADX92639) against ischemic neuronal death, while observing exacerbated neuronal damage following the application of an mGluR2 enhancer 2,2,2-Trifluoro-*N*-[4-(2-methoxyphenoxy)phenyl]-*N*-(3-pyridinylmethyl)ethanesulfonamide (LY487379) [51]. In contrast, an increasing body of evidence suggests that mGluR2/3 agonists indeed exhibit neuroprotective properties in several models of brain ischemia and experimental birth asphyxia [52–56].

The potential of mGluR2/3 agonists in neuroprotection was recognized over two decades ago. The initial observations of the neuroprotective effect of the selective mGluR2/3 agonist (-)-2-oxa-4-aminobicyclo[3.1.0]hexane-4,6-dicarboxylic acid (LY379268) applied shortly after HI in 7-day-old rats and during global ischemia in gerbils were reported more than 20 years ago [49, 57]. Later studies conducted by various researchers confirmed the neuroprotective effect of mGluR2/3 activation in ischemic conditions [11, 22, 58].

Further research has unveiled intriguing patterns in the efficacy of mGluR2/3 activation across different models and age groups. It has been demonstrated that the activation of mGluR2/3 in adult animals is more effective in providing neuroprotection in cases of global ischemia but also exhibits neuroprotective activity in a neonatal rat model of hypoxia–ischemia [49, 54, 55, 57, 59].

Two specific agonists have emerged as promising candidates for neuroprotection in hypoxic-ischemic conditions. LY379268, an mGluR2/3 agonist, shows a higher affinity towards mGluR2 modulation, while N-acetylaspartylglutamate (NAAG) acts as a highly selective endogenous agonist of mGluR3 [60]. Both agonists have demonstrated potent neuroprotective effects in experimental neonatal hypoxia–ischemia.

The mechanism underlying the neuroprotective effects of these agonists is gradually being unraveled. Hypoxia–ischemia triggers an increase in cAMP concentration, likely associated with dopamine release or PKC activation. Group II mGluRs are negatively linked to cAMP formation, and their activation reduces the HI-evoked elevation of cAMP, potentially aiding in the attenuation of inflammatory processes [61, 62]. Crucially, these agonists have demonstrated efficacy even when administered several hours after the hypoxic-ischemic event, suggesting a potential therapeutic window for intervention. LY379268 and NAAG, applied up to 6 h after HI, reduced ischemia-induced brain damage and neuronal loss in the CA1 area of the hippocampus and cerebral cortex in rat pups subjected to an experimental birth asphyxia model [55, 57, 59, 62].

To confirm that the observed neuroprotective effects were indeed mediated through mGluR2/3 activation, researchers employed a pharmacological approach. The inhibition of the neuroprotective effect of NAAG by a selective mGluR2/3 antagonist ((S)-a-Amino-a-[(1S, 2S)-2-carboxycyclopropyl]-9H-xanthine-9-propanoic acid (LY341495) confirmed the beneficial effect of mGluR2/3 activation [62].

Activation of mGluR2/3 also mitigated oxidative stress, a common feature accompanying HI and driving neurodegenerative processes. The application of LY379268 or NAAG shortly after HI significantly reduced the elevated levels of ROS in brain samples compared to untreated animals [58]. Notably, the activity of antioxidant enzymes (superoxide dismutase (SOD), catalase, glutathione peroxidase (GPx)), and glutathione decreased compared to untreated animals [59]. This suggests that the primary mechanism behind the observed effect likely involves the inhibition of glutamate release by neuronal mGluR2/3 and the contribution of glial mGluR3 in reducing extracellular glutamate concentrations.

The inhibition of apoptotic processes observed following the application of mGluR2/3 agonists is likely a consequence of the same mechanism [55]. The reduced expression of pro-apoptotic factors Bax and HtrA2/Omi, coupled with an increase in expression of anti-apoptotic Bcl-2, indicates that the activation of mGluR2/3 after HI may inhibit both caspase-dependent and -independent apoptosis. Our unpublished results show that the application of LY379268 up to 6 h after HI decreases the activity of both caspase-9, an initiator caspase crucial in the apoptotic pathway, and caspase-3, a key executioner caspase in apoptosis. However, an early publication by Cai and colleagues (1999) reported that LY379268 administered immediately after HI did not prevent the increase in caspase-3 activity and DNA fragmentation observed after HI in neonatal rats. This discrepancy necessitates further investigation.

It has been observed that HI diminishes the expression of neurotrophic factors, pivotal in neuroprotection. Studies have indicated that the activation of mGluR2/3 present in neurons, astrocytes, or oligodendroglia cells may stimulate the production of neurotrophic factors such as transforming growth factor-1 (TGF-1) [63], BDNF that inhibits apoptosis [64, 65], and glial-derived neurotrophic factor (GDNF) that can inhibit caspase-3 [66]. Therefore, the interaction between neurotrophic factors and the mGlu2/3 receptor signaling system may be important in preventing HI-induced brain damage. Early activation of mGluR2 and mGluR3 in hypoxic-ischemic conditions significantly reduced the HI-induced decrease in BDNF expression, which was beneficial for neuronal survival [55, 67, 68]. Conversely, the expression of GDNF decreased compared to untreated animals, with the authors attributing it to the protection of neurons by reducing glutamate release; however, this hypothesis requires further exploration [55].

Recent studies have also highlighted the involvement of mGluR2/3 in the induction of ischemic tolerance [59, 69–71]. While the reduction of glutamate release appears to be an obvious aspect of neuroprotection mediated by mGluR2/3 activation, the precise mechanism of this neuroprotection remains incompletely understood and appears to be multifaceted.

Neuroprotection achieved through the activation of mGluR2/3 receptors 24 h or 1 h before HI by the application of LY379268 or NAAG is primarily associated with the inhibition of glutamate release and the subsequent prevention of the development of neurodegenerative processes [59]. However, NAAG, in addition to activating mGluR3, also activates synaptic NMDA receptors containing the GluN2A subunit and inhibits extrasynaptic receptors containing GluN2B [72]. In the extracellular space, NAAG, unlike LY379268, is rapidly degraded by astrocytic glutamate carboxypeptidase into glutamate and N-acetyl-aspartate [73]. Therefore, the neuroprotective effect of NAAG applied 1 h before HI appears to be a combination of mGluR3 and NMDA receptors activation, while the effect of NAAG applied 24 h before HI most likely depends on mild NMDA receptors activation and the induction of ischemic tolerance [70]. Given the neuroprotective effects of mGluR2/3 activation in HI-induced brain damage, this experimental therapy is worth consideration as a novel treatment approach in the clinical management of birth asphyxia. However, further research is essential to fully elucidate the mechanisms involved and to translate these preclinical findings into clinical applications. Additionally, the potential long-term effects and safety profile of mGluR2/3 agonists in neonates need thorough investigation before clinical trials can be initiated.

These findings collectively suggest that targeting group II mGluRs, particularly with agonists like LY379268 and NAAG, could represent a promising therapeutic strategy for neuroprotection in neonatal hypoxic-ischemic brain injury. However, further research is imperative to fully understand the mechanisms involved and to translate these preclinical findings into clinical applications.

### Group III mGlu receptors

Group III mGluRs consist of four members: mGluR4, mGluR6, mGluR7, and mGluR8. These G-protein negatively coupled receptors, similar to group II mGluRs, modulate neurotransmission by inhibiting adenylyl cyclase, thereby reducing neurotransmitter release. This inhibitory function acts as a crucial feedback mechanism, aiding in maintaining the delicate balance between excitatory and inhibitory signaling in the brain. Group III mGluRs, primarily located presynaptically, function as autoreceptors [74–79], while those expressed postsynaptically are involved in the regulation of NMDA or GABA receptors [74, 78, 80–83]. Their expression and roles vary across brain regions such as the cortex, hippocampus, and cerebellum. This regional specificity makes them intriguing targets for neuroprotection in various neurological conditions, including brain ischemia, especially in neonatal contexts [84].

Group III mGluRs are widely distributed throughout the central nervous system, with distinct expression patterns for each subtype. This diverse distribution pattern suggests that each subtype may have specialized functions in different brain areas, contributing to the complexity of glutamatergic signaling regulation. In the cerebellum, mGluR4 is mainly expressed in the molecular layer and at parallel fiber synaptic terminals [78, 85]. The hippocampus displays a more intricate distribution pattern, with mGluR4, mGluR7, and mGluR8 expressed in various subregions, each contributing to the intricate regulation of hippocampal circuits [86, 87]. In the basal ganglia, mGluR4 and mGluR7 are expressed in the striatum, globus pallidus, and substantia nigra, suggesting their potential involvement in motor control and neurodegenerative disorders [88, 89].

The expression of mGluR4 increases during postnatal development from days P1 to P30, particularly in the cerebellum, where it becomes significantly detectable by P3. In contrast, levels in the thalamus and septal nuclei decrease, while striatal levels remain low but constant. Immunohistochemical studies indicate that strong mGluR4a signals in cortical layers II-VI begin to decline from P12, with significant levels still detected in the striatum at P12 [47, 90]. In the hippocampus, mGluR4 expression remains stable until P60. Meanwhile, mGluR7 exhibits high expression in the developing cerebellum by embryonic day 18 and peaks at birth in the neocortex [90, 91]. Notably, changes in the hippocampus demonstrate pronounced mGluR7 expression in the CA1-CA3 regions at birth, with shifts in proportions over time [91]. Furthermore, mGluR8 expression is significantly higher at the E16 stage compared to the adult brain, and it is found in various brain regions and the retina [92].

Despite the potential significance of group III mGluRs in neuroprotection, there is a scarcity of publications exploring their potential as a neuroprotective treatment in brain ischemia, with most focusing on mGluR4 and mGluR7. Furthermore, there is limited information investigating this potential in neonatal hypoxia–ischemia. Nonetheless, presenting the available information is crucial to stimulate further research in this area.

While the prevailing belief is that group III mGluRs do not play a significant role as a potential target for neuroprotection, they offer a promising alternative approach to mitigating excitotoxicity by modulating glutamatergic transmission rather than blocking it, as evidenced in various ischemia models [11, 93–95]. The activation of group III mGluRs initiates a downstream signaling cascade that regulates intracellular calcium levels, induces neuronal hyperpolarization, and reduces neurotransmitter release, creating a complex network of cellular changes that collectively contribute to neuroprotection [96, 97]. This modulatory approach allows for a more precise regulation of glutamate signaling, potentially preserving essential neuronal functions while providing neuroprotection [98–102]. Such nuanced modulation of glutamatergic transmission is particularly important in the context of brain asphyxia, where excessive glutamate release significantly contributes to neuronal damage, but complete suppression could hinder recovery. This delicate balance between neuroprotection and maintaining essential neuronal function is a key advantage of targeting group III mGluRs in ischemic conditions.

Research has shown that in the rat cortex and hippocampus, transient cerebral ischemia resulted in an increase in mGluR4 expression, particularly the splice variant mGluR4b, indicating the importance of these receptors for the proper functioning of these brain regions [103, 104].

It has been reported that (1S, 3R,4S)-1-aminocyclopentane-1,2,4-tricarboxylic acid (ACPT-I), another group III mGluR agonist, enhances the survival of cortical neurons after OGD. This effect was associated with the activation of mGluR4, rather than mGluR7 or mGluR8 [48]. ACPT-I also reduced OGD-induced neuronal cell damage, calpain activity, and glutamate release, demonstrating synergistic neuroprotection in combination with mGluR4 positive allosteric modulators. Moreover, ACPT-I reduced brain infarct volume, improved selective gait parameters and mobility in healthy rats, and in rats with essential hypertension subjected to the middle cerebral artery occlusion (MCAO) [105, 106]. Notably, when mGluR4 was deleted, the infarct volume increased by up to one-third after MCAO in mice [107].

In a recent study, Bossi and colleagues, using OptoGluNAM4.1, a negative modulator of mGluR4, demonstrated in experiments on rodent cerebellar slices that mGluR4 is exclusively activated in excitotoxic conditions such as simulated cerebellar ischemia [108].

The activation of mGluR4 by (R, S)-4-phosphonophenylglycine [(R,S)-PPG], another potent agonist of group III mGluRs, significantly improved the recovery of synaptic transmission in the CA1 region of hippocampal slices, likely through the activation of mGluR4 and/or mGluR8 [109, 110]. However, in vivo experiments did not support the beneficial activity of (R,S)-PPG in focal cerebral ischemia in mice or global cerebral ischemia in gerbils or rats, as it was found to be toxic and led to animal death [110].

mGluR7 also plays a role in the survival of cerebral neurons subjected to ischemic conditions. Activation of mGluR7 with N,N'-Bis(diphenylmethyl)-1,2-ethanediamine dihydrochloride (AMN082), a specific potent allosteric agonist, increased survival of OGD-treated mouse cerebral neurons [111]. Application of AMN082 attenuated OGD-induced necrotic cell death, inhibited OGD-evoked calpain activation, and reduced caspase-3 activity in the kainic acid (KA) model of excitotoxicity [111].

Pretreatment of rat hippocampal neurons with L( +)-2-amino-4-phosphonobutyric acid (L-AP4), a selective group III mGlu receptors agonist, showed significant neuroprotective effects in anoxia or NO exposure [112]. However, in neonatal hypoxia–ischemia, the application of L-AP4 shortly after the insult did not prevent damage to the ischemic part of the brain, although it was able to prevent HI-evoked elevation in cAMP concentration [62].

Overall, in vivo experiments have supported the hypothesis that the activation of group III mGluRs aids recovery after cerebral ischemia. These effects were typically measurable by the reduction of infarct size and improvement in behavioral tests in animals subjected to ischemia and subsequently treated.

The neuroprotective effects of group III mGluRs in ischemia seem to be mediated through various mechanisms. One key aspect is the reduction in glutamate release, achieved through the activation of signaling pathways involving G proteins and inhibition of adenylyl cyclase [113, 114]. The latter can lead to hyperpolarization by allowing K + efflux through small-conductance Ca^2+^-activated K^+^ (SK) channels and, specifically by activation of G(αβ), to the inhibition of voltage-gated calcium channels and stimulation of G-protein-coupled inwardly-rectifying potassium (GIRK) channels, further reducing neurotransmitter release [74, 101, 102, 115]. Additionally, the activation of group III mGluRs inhibits the cleavage of caspase-3 in cortical and hippocampal neurons, suggesting anti-apoptotic potential [116]. The primary mechanism of neuroprotective action of mGluR7 is also associated with inhibiting the ischemia-induced increase in the activity of calcium-dependent enzymes, calpains, which are also implicated in apoptotic cell death [111]. This indicates that group III mGluR subtypes may employ various neuroprotective pathways to prevent neuronal damage.

These findings underscore the potential of group III mGluRs as therapeutic targets in brain ischemia and neonatal hypoxia–ischemia. However, further research is essential to fully understand their role, particularly in neonatal contexts, and to translate these preclinical findings into potential clinical applications. The intricate interplay between different mGluR subtypes and their varied effects in different brain regions and under different conditions emphasizes the necessity for careful and targeted approaches in developing therapeutic strategies based on mGluR modulation.

*Conclusions and future perspectives *Scientific research aimed at discovering new, effective therapies for perinatal hypoxia has a long history. The deficits in oxygen and metabolic substrate delivery to the infant’s brain lead to a failure of ATP production, and disruption of neuronal cell function initiating a cascade of events that culminate in cell death. The first, and considered as the most important stage, is the release of excitatory amino acids, particularly glutamate, activation of its ionotropic NMDA receptors, and excessive influx of Ca^2+^ into neurons, triggering a cascade of intracellular events that result in cell death. Therefore, the main direction of research was to prevent the activation of NMDA receptors, which are considered to be key players in initiating excitotoxic processes, and exploring different methods to inhibit them. While this approach seemed logical, recent findings have challenged our understanding of NMDA receptor function in both physiological and pathological conditions. New data has revealed that the location of NMDA receptors determines whether their activation leads to pro-death or pro-survival signals. Specifically, in pathological conditions activation of synaptic NMDARs is neuroprotective, whereas activation of extrasynaptic NMDARs typically initiates cell death pathways [10]. Moreover, the discovery that NMDA receptor activation is a primary mechanism underlying synaptic refinement during development and that their inhibition can lead to neurodevelopmental disorders, has shifted research towards alternative ways of blocking these receptors [117–119].

Given that the activation of extrasynaptic NMDARs in hypoxic-ischemic conditions results from massive glutamate release and its diffusion beyond the synaptic cleft, limiting glutamate release appears to be a promising direction in the search for new ways to reduce brain damage induced by hypoxia–ischemia. The ability of group II and III metabotropic glutamate receptors to regulate glutamate release and uptake, and modulate the activation of NMDA receptors, makes targeting their activity a very promising idea. This modulation offers the potential to regulate the increased in the activity of NMDA receptors in hypoxic-ischemic conditions without disrupting synaptic transmission and the formation of normal neuronal connections in the immature brain.

**Fig. 2:**
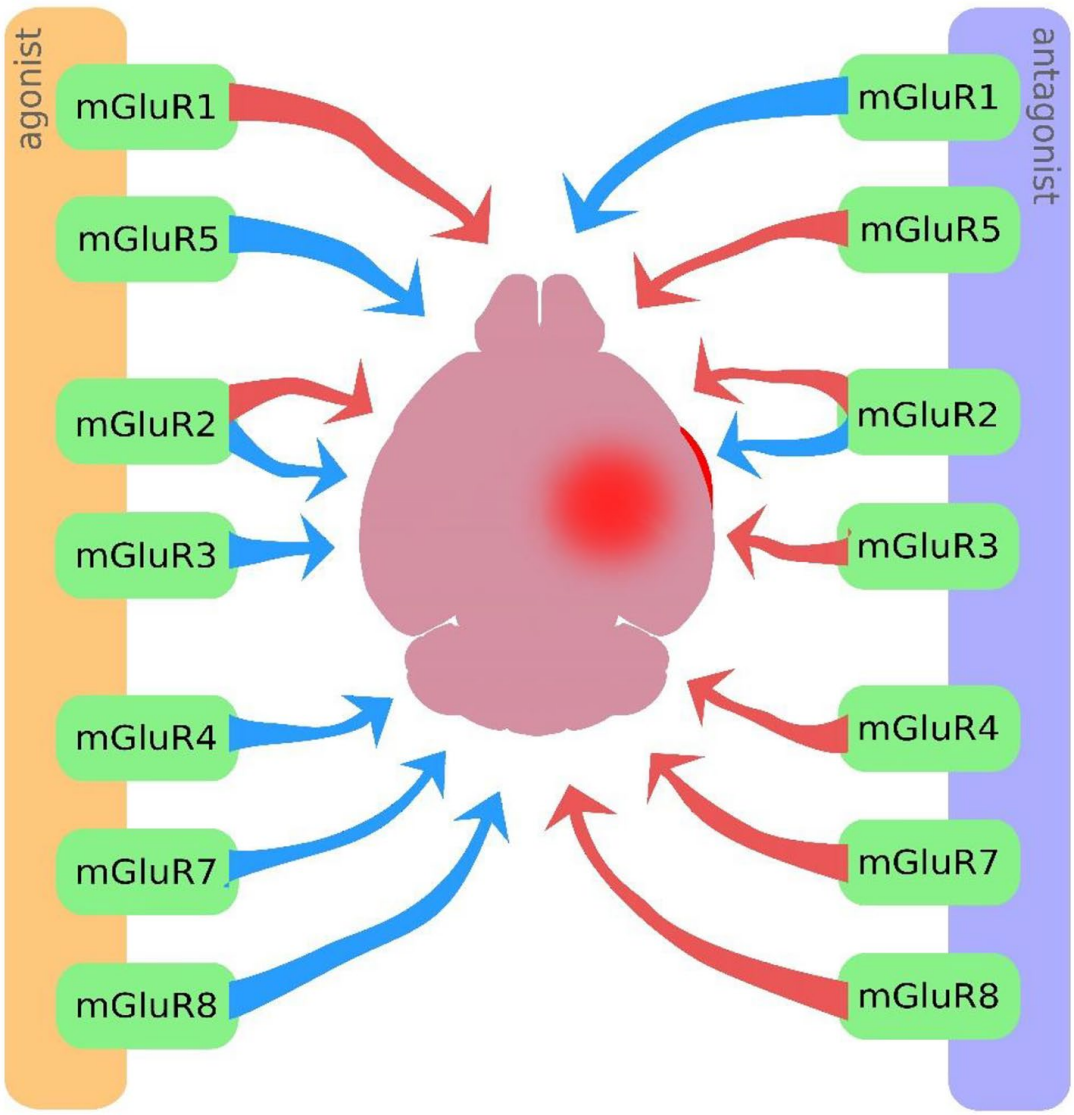
The impact of activating or inhibiting individual metabotropic glutamate receptors on their role in neuroprotective or neurodegenerative processes associated with hypoxia–ischemia. Blue arrows – neuroprotective effect; red arrows – neurodegenerative effect

Most experiments using metabotropic glutamate receptor agonists and antagonists in ischemic conditions have been conducted on cell cultures and adult animals, with fewer studies focusing on perinatal hypoxia–ischemia models. However, the analysis of published results allows us to determine how the activation or inhibition of individual mGluRs influences the development of brain damage after HI (Fig. 2). Results have shown that the application of antagonists [24, 26, 29] or even agonists [22–24, 31] of group I mGluR resulted in neuroprotection in adult animals with ischemia and neuronal cell cultures subjected to OGD. However, experiments have also revealed differences in the response to the application of group I mGluR agonists or antagonists between adult and immature animals under ischemic conditions. Particularly, the positive response to mGluR1, but not mGluR5, antagonist application after experimental birth asphyxia [29], highlights the importance of careful selection of compounds for their expected neuroprotective effects in developmental contexts.

Growing experimental evidence suggests a significant neuroprotective potential of group II and III agonists in the treatment of brain ischemia and birth asphyxia. mGluR3, for instance, not onlycontrols glutamate release but also activelyregulates extracellular glutamate levels by promoting its uptake by astrocytes [120]. Additionally, the activation of microglial mGluR3 has shown a protective effect, while the activation of mGluR2 appears to exacerbate neurotoxicity [121].

Since early experiments testing the neuroprotective potential of mGluR2/3 agonists in neonatal hypoxia–ischemia, the availability of selective agonists and positive modulators for both mGluR2 and mGluR3 has significantly increased. Many of these compounds have been demonstrated to inhibit brain damage and the development of molecular reactions leading to neurodegeneration induced by experimental brain ischemia in adult animals and neonatal hypoxia–ischemia. Importantly, it has been shown that the application of mGluR2/3 agonists both before and within the therapeutic window (up to 6 h) after experimental neonatal hypoxia–ischemia has a neuroprotective effect, primarily related to the inhibition of glutamate release [54, 55, 59, 62, 70, 122].

The glial-neuronal interaction mediated by mGluR3 appears to play a key role in neuroprotection, making the endogenous specific receptor agonist NAAG an object of scientific interest. Due to the rapid degradation of NAAG released into the synaptic cleft by glutamate carboxypeptidases II and III (GCPII, GPCIII), research has focused on inhibiting these enzymes. In experimental conditions, inhibitors of GCPII such as 2-(phosphonomethyl)pentanedioic acid (2-PMPA) and the thiol-based inhibitor 2-(3-Mercaptopropyl)pentanedioic acid (2-MPPA), have shown potent therapeutic effects in various animal models of diseases, including neonatal hypoxia–ischemia [123, 124]. However, classical GCPII inhibitors like 2-PMPA and 2-MPP do not penetrate the blood–brain barrier, posing a significant limitation for drugs intended to act within the brain. Moreover, although 2-MPPA was tolerated by healthy humans, subsequent tests revealed its immunotoxic effects, which were apparently not related to GCPII inhibition but rather to the thiol component [125, 126]. Nevertheless, the recent synthesis of non-thiol-based GCPII inhibitors with enhanced blood–brain barrier penetration capabilities, coupled with the observation that unlike in the case of group I mGluRs, no significant difference in the effects of agonists has been noted in experiments conducted on young or mature animals, may alter the landscape and provide GCPII inhibitors with an opportunity to advance to clinical trials.

A pattern of action similar to group II mGluRs characterizes the group III receptors, especially mGluR4 and mGluR7. These receptors are predominantly localized in presynaptic regions, and their activation also reduces glutamate and GABA release. Additionally, postsynaptically expressed mGluR4 and mGluR7 can modulate NMDA receptors [80, 81]. The beneficial effects of group III mGluR agonists have been demonstrated in numerous neurodegenerative disease models, including brain ischemia [96, 110]. While data on the use of group III mGluRs in animal models of neonatal asphyxia are limited, findings from OGD experiments on various neuronal cell cultures and hypoxia-hypoglycemia on brain slices, serving as in vitro models of neonatal hypoxia–ischemia, indicate the neuroprotective effects of selective agonists such as ACPT-I and (R, S)-PPG [97, 109]. The fact that group III mGluR agonists not only reduce brain damage in ischemic conditions but also improve motor functions in animals subjected to brain ischemia positions them as promising targets for future therapies [127]. However, these beneficial effects require validation in animal models of neonatal hypoxic-ischemic conditions.

Despite the promising potential of mGluRs as neuroprotective targets, the intricate and sometimes opposing roles of different receptor subtypes in various brain regions and pathological conditions necessitate a deeper understanding of their precise mechanisms of action. This complexity underscores the need for more focused research to elucidate the specific functions of each subtype in hypoxia–ischemia, considering their localization and developmental changes. Additionally, as most mGluR modulators have not progressed to clinical trials due to their undesirable side effects, there is a need for the synthesis of new, safe, and selective ligands for mGluRs to pave the way for the development of new therapies for the vulnerable infant brain at risk of complications following perinatal hypoxia–ischemia.

In conclusion, while significant progress has been made in understanding the role of mGluRs in neonatal hypoxia–ischemia, much work remains to be done. Future research should focus on developing more specific and safer mGluR modulators as well as on unraveling the complex interplay between different mGluR subtypes in the context of the developing brain. These efforts may ultimately lead to novel therapeutic strategies that can substantially enhance outcomes for infants affected by perinatal hypoxia–ischemia.

## Data Availability

No datasets were generated or analysed during the current study.

## Abbreviations

(RS)-PPG: (RS)-4-Phosphonophenylglycine, a non-specific agonist for the group III mGluRs
ACPT-I: (1S,3R,4S)-1-Aminocyclopentane-1,3,4-Tricarboxylic Acid, a non-specific agonist for the group III mGluRs
ADX47273: (4-Fluorophenyl)[(3S)-3-[3-(4-fluorophenyl)-1,2,4-oxadiazol-5-yl]-1-piperidinyl]methanone, positive mGluR5 allosteric modulator
AMPA: 2-Amino-3-(5-methyl-3-oxo-1,2-oxazol-4-yl)propanoic acid
BDNF: Brain derived neurotrophic factor
cAMP: Cyclic adenosine monophosphate
CHPG: (R,S)-2-chloro-5-hydroxyphenylglycine, mGluR5 agonist
DAG: Diacylglycerol
DHPG: (S)-3,5-dihydrocy-ohenylglycine (DHPG), agonist of group I mGluRs
EMQMCM: (3-Ethyl-2-methyl-quinolin-6-yl)-(4-methoxy-cyclohexyl)-methanone methanesulfonate, mGluR1 antagonists
GABA: Gamma-aminobutyric acid
GDNF: Glia derived neurotrophic factor
GPx: Glutathione peroxidase
HI: Hypoxia–ischemia
IP3: 1,4,5-Triphosphate
KA: Kainic acid
L-AP4: L-2-Amino-4-Phosphonobutyric Acid, a group III mGluRs non-specific agonist
L-AP4: L-2-Amino-4-Phosphonobutyric Acid, a non-specific agonist for the group III mGluRs
LY367385: ( +)-2-Methyl-4-carboxyphenylglycine, mGluR1 antagonists
LY487379: 2,2,2-Trifluoro-*N*-[4-(2-methoxyphenoxy)phenyl]-*N*-(3-pyridinylmethyl)ethanesulfonamide, mGluR2 enhancer
MCAO: Middle cerebral artery occlusion
mGluR: Metabotropic glutamate receptor
MTEP: 3-[(2-Methyl-1,3-thiazol-4-yl)ethynyl]pyridine, mGluR5 antagonist
NAAG: N-acetylaspartylglutamate, endogenous mGluR3 agonist
NF-κB: Nuclear factor kappa B
NMDA: N-Methyl-D-Aspartate
NRF2: Nuclear factor erythroid 2-related factor 2
OGD: Oxygen–glucose deprivation
PKC: Protein kinase C
PLC: Phospholipase C
ROS: Reactive oxygen species
SOD: Superoxide dismutase
